# Buffalo Academy of Medicine

**Published:** 1892-11

**Authors:** Wm. C. Krauss

**Affiliations:** Secretary


### BUFFALO ACADEMY OF MEDICINE.

*First Stated Meeting, September 13, 1892.*

Reported and edited by WM. C. KRAUSS, M. D., Secretary.

DeLancey Rochester, M. D., President.

### DISCUSSION OF LITHEMIA.

### ITS PHYSIOLOGICAL CHEMISTRY.

By WILLIAM C. KRAUSS, M. D., Buffalo, N. Y.

The food supply of the body is divided into nitrogenized, non-nitrogenized, and inorganic substances. The inorganic compounds comprise water, and salts, as chlorides, phosphates and carbonates of soda, potash, calcium, etc.

The non-nitrogenized foods comprise :

1. Fats, as cream, fat of adipose tissue, etc.
2. Carbo-hydrates, as sugar and starch.
3. Simpler organic bodies, as alcohol, vegetable acids, etc.

The nitrogenized foods comprise :

1. Proteids, or albuminous substances.
2. Albuminoids, as gelatine, chondrin, etc.
3. Simpler nitrogenized bodies, as creatin, lecithin.
4. Iron-containing compounds.

The inorganic principles do not undergo digestion and assimilation, but are at once absorbed by the system.

The non-nitrogenized principles, after being digested, are more easily oxidized in their end products than the nitrogenized elements, and this oxidation once under way does not cease until it is completed. In case an increased quantity of these carbo-hydrates are present in the system, the oxygenating capacity of the blood will become exhausted, and, as a result, the more difficult nitrogenous compounds are imperfectly transformed. From the oxidation of the carbo-hydrates we obtain heat, energy, lubrication, and elements producing rotundity of form only. The excretions are water and carbon dioxide.

Among the nitrogenized principles the most important are the proteids; in fact, they are the most important substances that occur in animal or vegetable organisms, being invariable and constant constituents of protoplasm. It is difficult to give a logical definition of the proteids. We can only say what they are composed of, and how they act in the human economy. Garrod describes them as "highly complex and uncrystallizable compounds of C. H. O. N. & S., occurring in a solid viscous condition, or in solution, in nearly all the solids and liquids of the body." Although differing from each other in physical and, to a certain extent, even in chemical properties, they, however, all possess certain chemical reactions, and are united by a close genetic relationship. The proteid constituents of the animal body are derived from vegetables, either directly or indirectly, through the body of another animal. The chief animal proteids in food are the myosin of flesh, the casein and the albumen of milk, and the proteids of egg and blood. The chief vegetable proteids are gluten, vegetable myosin, and other vegetable globulins. The animal proteids are more easily digested than those of vegetable origin. The action of the juices of the alimentary canal, especially the gastric and pancreatic, upon the ingested proteids, is to convert them into peptones, which are then absorbed. The peptones are reconverted into proteids, and these require considerable oxygen to transform them before they can be assimilated or made part of the living organism. This process is commonly termed tissue growth, tissue repair, or building up, but alongside of it there is a destructive process going on in the cells—a " tissue waste," or breaking down. These two forces are designated metabolism. The result of the constructive metabolism, or anabolism, of the proteids means nutrition to the whole system— the production of heat, energy, muscular and glandular activity, rotundity, and constituents for the formation of the ferments. The result of destructive metabolism, or katabolism, is the formation of the various oxides, carbon dioxide, water, and certain not fully oxidized products—as uric acid, urea, etc. The discharge of the products of destructive metabolism by the air, urine, sweat, and feces, constitutes excretion. In a normal healthy body, supplied with a sufficient quantity of food, drink and exercise daily, the metabolic changes take place according to Nature's inflexible laws ; waste products, the result of oxidation, are thrown off, and the system thus nourished and reinforced is able to hold its own in the struggle for existence.

On the other hand, in a body perhaps handicapped by some hereditary taint, where over-eating and over-drinking are indulged in with the consequent sedentary habits, oxidation changes of the body are lessened, uric and oxalic acids are formed to excess, and accumulating in the system, produce such derangements as calculus, gravel, gout, sclerosis of the various organs, toxic conditions.

Perhaps it would be well to study uric acid somewhat closely and see wherein lay its obnoxious qualities. It is composed of five atoms of carbon, four each of hydrogen and nitrogen, and three of oxygen. It crystallizes in colorless rhombic rectangular plates, or in rectangular prisms. In striking contrast to urea, it is a most insoluble substance, requiring for its solution 1,900 parts of hot, and 15,000 parts of cold water. It is slightly soluble in ether and alcohol. It is dibasic, forming normal urates and acid urates. The most abundant urate obtained from the human urine is the normal urate of soda. Small quantities of those of potassium and calcium also occur. The urates, like uric acid, being insoluble, precipitate when they are found in excess in the urine. The urates of lithium and potassium are more soluble than the urate of soda, and, hence, the lithium and potassium salts are often administered to counteract the insolubility of the urate of soda. The pinkish deposits of urates often called lithates, or brick dust deposit, which are found when a concentrated acid urine stands in the cold, are composed mainly of the acid urate of soda. This deposit occurs chiefly when the urine is concentrated, as after violent exercise and consequent profuse sweating. It also occurs in concentrated urine of fever, after indigestion, joint inflammations, in certain heart and lung diseases, cirrhosis of the liver, and in catarrh of the bladder. Hence, the appearance of a sediment of urates does not necessarily imply an increased formation of uric acid, but is generally due to an increased concentration of urine.

That uric acid has no particular function in the human urine is attested by the fact that although it is a vehicle for the elimination of nitrogen, urea, which is much more soluble, is therefore better fitted for this work. In birds, reptiles, and in some invertebrates, in which the urine is solid instead of liquid, uric acid is the principal nitrogenized constituent.

The quantity of uric acid present in the urine of a healthy man varies from seven to ten grains daily. It is reduced during hunger, increased after meals. The quantity excreted on an animal diet is greater than on a vegetable diet. Free uric acid in crystals occurs in the urine of persons with a uric acid diathesis, also often in diabetic urine. It is greatly increased in the urine of leucocythemia, and diminished in most chronic diseases. It is especially diminished in gout, where, according to Garrod, it accumulates in the blood and tissues. Haliburton thinks that because uric acid is found in the urine at least sixteen out of twenty-four hours, there must be some ingredients present in the urine which prevent it from precipitating. These inhibitory ingredients are thought to be the mineral salts and the pigments of the urine. The conditions which tend to accelerate the precipitation of uric acid, as in the formation of concretions and deposits, are: (1) high acidity; (2) poverty in mineral salts; (3) low pigmentation, and (4) high percentage of uric acid.

Whether uric acid exists in the blood or not, is a mooted question. Porter believes it cannot, because it would immediately join with the alkaline compounds and become a urate of some form. Roberts is sure that uric acid is not in the free state, because it is chemically impossible for uric acid to subsist in the alkaline blood serum without entering into combination with the bases contained in it. Garrod believes that uric acid does exist in the blood in a free state, and has proven it by various tests. His experiments are open to the criticism that many active chemical agents were necessary in the test before any signs of uric acid were evident. His opponents declare, therefore, that the acid was probably manufactured artificially during the different stages of his experiments. Von Jaksch[1], who has lately examined the blood of 109 individuals, found no traces of it in nine healthy persons, nor in cases of typhoid fever, nervous affections, diseases of the liver and gastro-intestinal tract, except when anemia coexisted. On the other hand, it was present in those diseased processes in which oxidation was disturbed, either directly, as in affections of the lungs, as pneumonia, or indirectly, as in anemia, in which the oxygen-carriers are deficient. Haig has no doubt as to the presence of uric acid in the blood, and affirms that the amount varies according to the degree of alkalinity, and all circumstances which increase this are associated with an increase in the amount of uric acid.

### SEAT OF FORMATION.

Landois and Sterling believe that it is formed outside the kidneys. Halliburton has two theories as to its formation. One is, that it is formed, like urea, in the other tissues, especially the liver and spleen, and is excreted by the kidneys. The other is, that the kidneys are not only the seat of excretion, but also that of formation of uric acid. Minkowski regards the chief feat of formation to be the liver, the result of synthesis of ammonia and lactic acid. Horbaczewski believes the spleen to be the organ of uric acid formation. Garrod holds that the kidneys are concerned, not only with its secretion but with its formation. Ebstein, on the other hand, thinks that it is chiefly produced in the muscles and bone marrow. Cadge, Riggs, Granville and Kirke are inclined to the theory that, as the hepatic cell has the power to form bile, so the epithelial cell of the renal tubule, by the metabolic activity of its protoplasm, has the power to produce some, if not all, of the urea and uric acid found in the urine. My own belief would coincide with Ebstein's, that it is formed largely in the muscles, because here metabolism is most active.

The dangers of an excessive amount of uric acid and its compounds in the human system seem to be:

First, on account of the great insolubility of this acid, it is prone to precipitate either in the bladder as gravel and calculi, in

1. Osler, p. 731.

the joints, kidneys, on the heart valves, vocal cords, about the cartilages of the ears, eyelids, etc., as gouty concretions, or along the tendons, as in some rheumatic troubles.

Second, owing to its irritative qualities, a slow form of sclerotic development takes place in the various organs, especially in the arteries, kidneys, liver, and central nervous system, calling forth such affections as arterio sclerosis, contracted kidney, and system diseases of the spinal cord.

Third, on account of its large contingency of nitrogen, it produces toxic conditions, particularly of the nervous and digestive systems.

To designate a certain group of vague, bastard symptoms, thought to be the result of some liver disturbance, Murchison suggested the term lithemia. Since then, symptoms arising from all organs and tissues of the body have been placed in the same category. Flint had previously described the same clinical picture under the head of uricemia. More recently Von Jaksch designated it as uricacidemia. Gray suggests lithoidosis for this condition, while those of us unable to keep pace with such names call it simply uric acid diathesis, or if to puzzle the layman, lithiasis, or lithemia. Tersely stated, then, lithemia means deficient oxidation of the proteids, imperfect or retarded metabolism, and the excessive formation, accumulation, and absorption of the katabolins, especially uric acid.

If I have failed to make my subject clear, I have the satisfaction in knowing that there is authority upholding me. Let me quote from Osler's recent work on the Principles and Practice of Medicine, page 733: "In the present imperfect state of knowledge, it is impossible, with any clearness, to define the so-called pathology of uric acid diathesis."

382 Virginia Street.

---

### CLINICAL ASPECTS OF LITHEMIA.

By DR. JOHN H. PRYOR, Buffalo, N. Y.

In compliance with the suggestion that discussion should be limited to those pathological disturbances described by Murchison and others as mild or irregular forms of gout, I shall avoid any consideration of true gout, and accept the term lithemia as applied to a group of morbid phenomena, supposed to be allied to gout in origin, but distinguished from acute or chronic gout by the absence of the classical signs of that disease. The literature of the subject is extensive and, at present, it has become the fashion to carelessly include many puzzling morbid conditions previously belonging to the no name series of disease, as evidences of lithemia. In a general way, the condition is recognized as similar to gout by its manifestations and assumed causation, but receives a different name to mark the contrast in clinical appearances. A suspiciously large number of slight departures from health are said to be caused by the presence of an unknown quantity of uric acid in the blood. Obviously, this common conclusion should depend upon the detection of the poison in the blood and the knowledge of a direct relation between its morbid action and effects. Unfortunately, little has been attempted or accomplished by scientific investigation in this direction. That the amount of uric acid in the blood is increased during an attack of so-called lithemia, has never been properly established. Haig and other enthusiasts assume its presence with remarkable indifference to scientific accuracy. Jaksch and Pfeiffer have failed to find traces of it in the conditions described as lithemia and rheumatism, and have observed the most notable increase in pneumonia, leukemia, and disorders associated with cyanosis, where carbon dioxide had accumulated in the blood or oxygen was diminished. Investigation has, at present, not been sufficiently extensive to justify the conclusion that the term lithemia rests upon a scientific basis. The belief, therefore, that a group of complex morbid phenomena are due to a certain toxic and irritating product, whose presence in the blood cannot be determined, must rest largely upon the results of clinical observation.

Careful study of the older writers reveals the pertinent fact that inquiry did not extend beyond the assumption of a clinical resemblance between the evidences of gout and lithemia and the probability of a common theory of causation. Whether Garrod's theory of the causation of gout is correct may be still open for controversy, but the apparent association of uric acid with destructive lesions forms striking proof entirely absent in lithemia. If it be admitted that uric acid may be present in the system in quantity which for some reason eludes detection, and yet sufficient to cause the condition called lithemia, we are prompted to ask, from a clinical standpoint, are we at present able to state authoritatively that any of the accepted disturbances attributed to uric acid are due to that agent alone? The toxic effects of uric acid have never, so far as I know, been experimentally demonstrated. A poison introduced into the system stimulates or depresses functional activity, according to the dose, and presents a picture quite characteristic and invariable.

Allowing a large field for idiosyncrasy, etc., we find that no other toxic element is assigned so huge and varied a *rôle* as the obscure agent, uric acid. Poisons rarely act alone, but produces mixed toxemia by disturbing functions and their interdependence. Uric acid is probably the result of interrupted or incomplete metabolism, and the symptoms of toxemia are usually manifest during a period of mal-assimilation. Progress in pathological chemistry is rapidly explaining the important part played by leucomaines and ptomaines at this time, and we have yet to learn how to differentiate between their manifestations and those often ascribed to lithemia. Many of the digestive disturbances described as lithemia in origin by Murchison and others are now considered as symptoms of anto-infective toxemia, presumably complex. Some stress is laid upon the augmentation of urates in the urine, and deductions have been drawn in the laboratory which are, as usual, not borne out clinically. Some authorities claim that an increased amount of urates or uric acid in the urine is evidence of a pathological surplus in the blood, and others take the opposite view.

One fact must remain undisputed. At times the urine is loaded with a sediment of lithates which is indicative of disturbed digestion and faulty metabolism. What relation, then, does lithuria, evanescent or chronic, bear to lithemia ? Frequently the two are not associated. It is not uncommon to diagnose tentatively certain ailments, as the so-called lithemic headache, and watch in vain for the appearance of uric acid or urates in increased amount in the urine. It must not be forgotten that the amount of urinary water passed in a given time may lead to false conclusions in estimating the amount of sediment. We find lithuria most pronounced in conditions characterized by pyrexia and lack of oxidation. Particularly pneumonia, rheumatism, acute diseases with high temperature, and wasting diseases. What proportion of the symptomatology of these affections is contributed by uric acid cannot be defined, but we rarely perceive the form of toxemia which cannot be otherwise explained. As an example, the toxines of pneumonia, and those probably active in other acute affections, accompanied by fever, may produce the effects previously assigned to lithemia. There is no room for doubt that lithuria may exist with no discomfort whatever, and sometimes the complaint is only of local irritation. When lithuria is coexistent with or follows an attack of illness, I should accept it as an indication of perverted function, the symptomatology, however, to be studied by considering other factors in disease. After some study of this subject, one is apt to reach the conclusion that the term lithemia has a vague meaning, and is much misapplied ; that the accurate diagnosis of lithemia, as we understand it, is impossible ; that we are in ignorance concerning the toxic action of uric acid in these ill-defined cases, and that it is difficult or impossible to decide that any phase of toxemia is lithemic in nature. But the literature abounds with myriad forms of diseased action ascribed to this causative agency, and we have unwittingly assumed the anomalous position of referring frequently to a condition recognized clinically as lithemia when the diagnosis is incapable of scientific verification. Admitting that diagnosis must be largely conjectural, we are led to ask if the evidences of lithemia are sufficiently distinctive to be grouped under one head, and what particular clinical aspects belong exclusively to this condition ? In the course of time and experience, clinical observers have marked a relationship between certain signs and symptoms and gout. They have come to be known as premonitory or incipient indications of that disease, and belong, when properly identified, to its natural history. The error is revealed in the attempt to extend the number of these manifestations, and collect many indistinct and dissimilar conditions under the term lithemia. Perhaps the mistake may not be as apparent in England as in the United States. Here gout is rare, while lithemia seems to be very common. I am inclined to believe that an English specimen of lithemia must be of a more vigorous type than those displayed here.

Most of our ideas upon this subject have been imported and accepted without reflecting upon the essential differences in people, mode of life, and surroundings. The plethoric type is rare in this country, while the anemic is common. Both, however, are usually examples of indigestion, one suffering from non-elimination and the other from incomplete preparation of food for assimilation, and neurasthenia. An American writer finds great difficulty in distinguishing what is called lithemia from neurasthenia and the manifold forms of indigestion. Some who diagnosticated lithemia with facility some years ago, are now wondering how they did it. To express a personal opinion, I believe that most cases diagnosticated as lithemia, not gouty in character, properly belong to the domain of neurasthenia, overwork, and disturbance of digestion.

If lithemia were regarded as a symptom or secondary manifestation of faulty metabolism, its nature might be better appreciated and the danger of mistaking cause for effect might be removed. Still, the fact that one chemical compound in the interrupted chain of the chemistry of nitrogenized material is, in all probability, not alone responsible, will leave a puzzling problem for further investigation. In discussing the clinical features of lithemia, the question of the influence of heredity seems to be of great importance, especially from a diagnostic standpoint. A person is said to inherit the lithemic diathesis. These patients are principally afflicted by incompetent digestive organs, which may rebel early or late in life. Any excess or errors in diet may produce a disturbance of digestion, sometimes characterized by a heavy urine and other symptoms of lithemia. They are usually unable to eat sweet or starchy foods, or drink alcoholic stimulants, without unpleasant consequences. Sometimes the condition is present in infancy or childhood, and is displayed by the concentrated urine loaded with lithates. Often, however, the urinary water is diminished in quantity, and changes in appearance after a free supply of water or the administration of a mild diuretic. As an indication of a diathesis, I consider this occurrence in childhood as much exaggerated. The inherited tendency is often associated with neurasthenia, melancholy or irritable temperament, and belongs most frequently to intellectual people or brain workers, the offspring of parents who have indulged in excesses of vicious enjoyments or mental work. The tendency may be acquired by sedentary life, bad surroundings, improper diet, and abuse of the digestive organs. Often it seems to be of nervous origin, following much mental labor or distress, and lack of proper rest. The diathesis, then, seems to be a liability to develop imperfect digestion and assimilation, and the attacks may be frequent, ephemeral, more or less constant, or at long intervals. The common time is during the Winter or, more especially, the late months when Spring is approaching. It has been surmised that uric acid accumulating during the Winter is stored up in the system somewhere and escapes later into the circulation to be eliminated. It is more probable that trying weather, lessened exercise, social pleasures, and greater mental exertion, tend to lower the general health and resistance, and finally invites functional derangement. Weather and climate certainly bear a decided causative relation, and patients are apt to suffer more during a rainy, cold season, or when humidity is high.

Attacks may be averted or shortened, many times, by change of climate and scene. They are announced as general or local, acute or chronic. Usually they are not persistent, but follow periods of good health. The interval depends much upon the habits of the individual and his caution in keeping general health up to the standard. In acute attacks, the symptoms are those of indigestion and toxemia, as a rule. After considerable study of this variety of lithemia, I have reached the conclusion that it cannot be distinguished from other forms of acute indigestion, and especially that condition which has been called a bilious attack. Some suffer readily from an error in diet who never display other symptoms of lithemia, and the true nature of so-called lithemic dyspepsia will only be solved by a more careful examination of the steps in digestion. The other manifestations are confined almost entirely to the nervous system, mucous membranes, and skin. Neuralgia generally affects the intercostal and scrapulæ nerves, and seems to follow the rules laid down by Austie. I do not recall having seen a case where other conditions, such as nerve tire or hunger, did not assist in explaining the causation. Sciatica and lithic acid headache, in the form of migraine, are occasionally associated with other signs of the gouty vice called lithemia. In view of our lack of knowledge of these particular affections, such a belief is not surprising. I recall a case of sciatica, diagnosticated as distinctly lithemic (how I know not), which refused stubbornly to yield to any treatment based upon that idea, but recovered after rest, forced feeding, and electricity. Myalgia is cited very frequently as lithemic and, again, rheumatic. Perhaps the most marked feature of the diathesis in a mild form is the tendency to congestion and inflammation of the mucous membrane, particularly the respiratory and urinary tract. Certain individuals take cold very easily. Many times the previous history is an account of loss of sleep, excesses, late dining or irregular meals, but we meet patients who have observed the rules of health and still suffer from subacute exacerbations in which it is impossible to effect a cure without a change of climate. Laryngologists have described a form of pharyngitis which resists local treatment and is evidently a local manifestation of some general disturbance. It has been called gouty or lithemic pharyngitis. Alkalies are most beneficial, but their employment is based upon clinical results. The urine, in these cases, rarely shows any departure from the normal, unless the affection is accompanied by fever. Retraction and subacute inflammation of the gums are explained as lithemic in origin. Aside from those cases caused by bacterial infection, there remain others apparently due to acid secretions and malnutrition. To accept lithemia as a cause, is to believe that uric acid encourages malnutrition and decay and assists in preventing proper assimilation. In regard to the complications affecting the urinary apparatus, there are two positions stoutly maintained.

That uretritis may be excited by the presence of uric acid in the blood, according to the theory offered to explain the peculiar inflammations of the upper air-tract, or by the direct irritation of uric acid in an acid urine. Phosphaturia and evidence of a preceding gonorrhea must be excluded. Undoubtedly, cases occur which are not incited by other causes so far as we know. During the past three years I have seen several cases of purulent uretritis, quite severe and prolonged in character, where repeated examination for gonococci and other forms of bacteria have failed to detect them, and slight attacks of simple uretritis are not uncommon. Occasionally, uretritis occurs during attacks of typhoid fever, pneumonia, rheumatism, and other diseases where the urine is scanty, acid, and loaded with sediment. Gonorrhea in a gouty patient is usually prolonged and intractable. For the past two years I have had under observation a patient who is attacked once or twice each Winter with tonsilitis and much mental and physical disturbance. The fever runs high, and after two days the urine is loaded with urates to a remarkable degree. Then follows a violent inflammation of the neck of the bladder and urethra. I have also seen one case where uretritis, non-specific in character, always accompanies a cold. There is an undoubted relation between the so-called gouty sore throat, congestion of the larynx, bladder and urethral troubles, and affections of the skin, particularly urticaria of the miliaria form, and eczema. They are sometimes associated with the gouty vice, and are evidently due to some irritating or toxic element in the system, which either acts directly or by affecting nervous mechanism. These cases, however, accompany exhaustion, indigestion, and general impairment of function, at times, and only yield to efforts directed to increase general tone. It is well to eliminate, particularly, congestion and relaxation of the mucous membrane, which is associated with vicious habits and consequent depression of general tone. Again, cases apparently lithemic do not respond to antilithemic treatment, and it must be remembered that toxic properties not understood may be actively at work, or the condition is one of many phases interblended. Of the chronic forms I shall allude only to endarterits and the cirrhotic kidney. Calculi and gravel are better considered at another time. Patients undoubtedly gouty develop these lesions, and it is probable that uric acid is one of the poisons which produces them. This has not been definitely established, however. Unless the patient is distinctly gouty, we are not in a position to affirm that connective tissue hyperplasia is often associated with, or the result of, so-called lithemia. There are serious objections to the term gouty kidney. Enderitis and the cirrhotic kidney do not follow or accompany the lithemic state in a sufficient proportion of cases to warrant the fear of those alarming terminations. Clinical experience, attention to history, and *post mortem* observation fail to support the close connection which some authors try to establish, and to recognize, clinically, the causes of these affections is extremely difficult while their pathology is so little understood. That lithemia produces the vaso-motor disturbance in the form of high tension which sometimes precede these lesions, is purely conjectural. If it be believed that vicious matter and waste products bear an etiological relation to these affections, a method must be devised to distinguish the part played by uric acid and define its importance as a member of a group constantly growing larger. You will perceive that many clinical features of lithemia, mentioned in the literature, have been omitted. I have selected only those which were capable of some definition. Some points may bear repetition in dealing with the question of diagnosis, which hinges largely upon a discrimination between our understanding of gout and lithemia. They are almost hopelessly confused by authorities. If we regard lithemia as a suppressed or obscure form of gout, there is little need of an additional term. The controversy will then arise as to the propriety of including so many conditions under the head of the protean aspects of gout, with so little clinical or scientific evidence to warrant it. The term lithemia, used in a separate sense, may promote utility, but discourages inquiry. Considering then, lithemia as a distinct group of affections, allied to gout in an inexplicable way, we must distinguish them from the manifestations accompanying true gout and occurring with the paroxysms or during the intervals between attacks. Under the term lithemia are grouped the obscure conditions not distinctly gouty, and they are so distinguished by writers. Yet the diagnosis is made to depend almost entirely upon the presence of the gouty diathesis and the history of gouty vice. Personally, I am very skeptical in regard to the diathesis as displayed in connection with those manifestations attributed to lithemia, and do not believe that much can be gained by discussing an ill-defined thing, called diathesis, which is a combination of the effects of a predisposition, and cannot be recognized until they are perceptible as morbid phenomena of the disease itself. We conclude that a person has a tendency to certain attacks after he has demonstrated it, and to decide upon the cause demands a consideration of the circumstances which might promote such morbid action. The more closely the evidences of lithemia resemble those of gout, the easier the diagnosis, and it is difficult to state that a patient is lithemic unless he is distinctly gouty. At present, the diagnosis of lithemia is necessarily a matter of individual opinion, and there is danger of viewing this subject through a pinhole and losing sight of the pertinent fact that the condition, when capable of recognition, should be regarded as a symptom. Of late it is becoming apparent that the examination of the urine has been assigned too important a place as an aid in diagnosis. Haig, in his recent book on Uric Acid, which is chiefly interesting and amusing as an illustration of careless statements and plausible theories, insists upon the importance of determining the relative quantities of uric acid and urea, a suggestion which seems to have merit, but subsequent observations have failed to secure his novel results.

Treatment designed to assist in eliminating uric acid consists of the use of alkalies and alkaline waters, principally lithia salts and phosphate of soda. Recent investigations seem to show that the action of the lithia salts, as solvents of uric acid, has been very much overestimated. The consensus of opinion at present seems to be that soda salicylate is the most efficient agent. Drinking large quantities of water to flush out the system and dilute the urine, promoting free perspiration and proper action of the bowels, are important. One of the best cathartics for the latter purpose is calcined magnesia alone, or after a cathartic pill. Further production should be checked by denying food, or supplying a diet easily digested, which will depend largely upon the patient's lack of appetite or craving. Plans of diet have been devised to prevent attacks of lithemia, formed upon the theory that uric acid is elaborated because the last step in preparation for elimination is omitted, owing to the absence of sufficient oxygen to transform it into urea. Two diets are recommended; one composed of albuminous food and the other of carbo-hydrates. To state the claims of each seems unnecessary, as this matter has been much discussed. Clinically, we find that the diet must be suited to the case, and we cannot follow strictly any absolute rule. A mixed diet and avoidance of obnoxious materials, with the caution to eat sparingly, is more often rational. Proper and systematic exercise is of most emphatic importance. Advice in this direction is, in many cases, all that may be desired. The best method of administering oxygen is to increase the depth and number of respirations by exercise in the open air, and assist elimination at the same time. Patients can obtain all the oxygen required if there are enough red blood corpuscles to receive and carry it without inhalations. There is no sure method of treating lithemia, or the conditions which resemble it. One thing is certain: it rarely exists alone, and indigestion, neurasthenia, and other associated conditions should be remedied according to the particular indications, which differ in each case. More explicit directions for treatment are readily found in the text-books, and their value will vary with the ideas of the reader. In this rough paper I have endeavored to touch some of the perplexing questions which are of clinical interest, and have expressed with pardonable frankness, I trust, some opinions and conclusions which are at variance with those generally held. Perhaps they may be regarded as extreme, but they are intended to arouse discussion.

---

### DIGESTIVE DISTURBANCES IN LITHEMIA.

By CHARLES G. STOCKTON, M. D.

In May last, I had the honor of presenting this phase of the subject of Lithemia before the Association of American Physicians, at Washington, and also before the Central New York State Medical Association, at Syracuse, and, therefore, I cannot promise the Buffalo Academy of Medicine anything on the subject which already they may not have read or heard.

The position taken on the relations existing between the digestion and lithemia may be expressed, perhaps, better by quoting the definition given in a previous paper, namely: Lithemia is an auto-infection occasioned by imperfect primary digestion, usually arising in the stomach; and the failure of this organ is the result of too feeble innervation in those whose lives are relatively sedentary, and whose drafts upon the nervous system are so great as to induce appetites proportionately too large for the physical activity of the organism.

As to this definition, it should be explained that it is conceded that those who suffer from a gouty diathesis, more frequently than others, may be affected with lithemia under the given conditions.

This statement I desire to emphasize, that lithemia is not gout, either acute or latent, typical or atypical, but it is an auto-infection, an auto-intoxication, and the most offending substances that the lithemic has to contend with are by no manner of means uric acid and urates. Just what the peculiar toxic substances may be which give rise to those curious symptoms, referable to almost every function of the body, it is not possible to say, notwithstanding the excellent work done in this direction by Brieger, Bouchard, Gautier, Vaughn, and others.

To assume that this toxemia, which we call lithemia, comes alone from imperfect primary digestion, would be going too far. To begin with, there must be the tired nervous system, the diminished muscular and glandular activity which are so commonly seen in the brain worker.

With this condition goes imperfect oxygenation, and still the patient does not suffer from the marked symptoms of lithemia until the primary digestion fails.

I am aware that this position is a somewhat novel one, and that it is likely to be attacked, but it is a position which I have reached after a somewhat long and careful study of this subject, and after experience which has led me to change the opinion which I formerly had. The reason that the primary digestion has not been held more responsible for the lithemic attacks, is because the patient frequently makes no complaint of indigestion ; in point of fact, one may suffer most seriously from gastric or intestinal fermentation, resulting in the production of toxines of a more or less disturbing character, without producing any distress which the patient refers to his digestive tract. It has been found that victims of lithemia are relieved by an albuminoid diet in many instances, and yet he would be a hardy prescriber who would advise his gouty patients to live upon meats. Now, the reason that albuminoids so often agree with the lithemic is because these substances are less likely to give rise to fermentation in the alimentary canal.

In some instances, albuminoids do not relieve the lithemic, and then it will be discovered, upon precise examination of the gastric contents, that the proteids are not readily digested, and that they undergo putrefactive changes, and thereby lead to toxemia. In such instances the withdrawal of albuminoids is followed by a relief of symptoms. The proper treatment of lithemia, therefore, should be the rational management of the digestive apparatus. By this it is not meant to imply that the local treatment of the stomach, by medicaments, lavage, electricity, or other methods, should necessarily be resorted to, for very frequently it is found that the best management for indigestion is to send the patient out of doors, in some dry and sunny place, and require him to exercise in some way, which will lead to exhilaration of the nervous system and stimulation of the lymph and blood currents. To be dogmatic, I should say that "houses" produce most cases of indigestion. Do away with houses, and men must become active where they are now sluggish, and slow where they are now active. It means that they must use their muscles and their lungs, and not so much their nerves. This, it will be seen, is precisely the line of treatment which experience has shown to be the most useful in the treatment of lithemia. Lithemics all recover if they lead active lives in the dry, sunny, and open air, and so, also, do dyspeptics, unless the digestive organs have become so dilapidated by long exhaustion that structural disease is brought about.

Some one may remind me that gouty patients also improve by this manner of living, and Abernethy's epigram may be quoted, that the cure for gout is to "live upon a shilling a day, and earn it." Yes, it is undoubtedly the proper management for gout. By improving oxygenation we lessen the amount of uric acid in the blood, and gout is prevented.

I am quite prepared to believe the statement which I once heard made by Dr. Gardiner, of Glasgow, that the appearance of gout in Scotland followed the establishment of the carriage as a common convenience, but I should like to add to this, that the more common use of dark meats has had a share in producing this disease, and that while Gardiner's explanation of the cause of gout is true, Abernethy's treatment of it is equally true.

Now, in this country, it seems to me that the treatment of lithemia should consist, first, in requiring a liberal amount of pleasing exercise in the open air, in a dry climate and in the sun. Second, to restrict the diet to such articles as are most easily digested by the individual under treatment. There should be no special rules save that those foods which disagree should be avoided. Third, the selection of these foods is not to be determined by the sensations of the patient alone, but if the symptoms continue, the stomach contents should be withdrawn and examined with sufficient frequency to discover how digestion is progressing, and what foods are giving rise to the disturbance. It will rarely be found necessary to resort to other medicaments than those required for correcting a slow digestion, and in this way the amount of alkalies and purgatives which will be required to make elimination comfortable and successful will be very small. Where it is impossible to observe these rules, where, for instance, patients are unable to exercise, or unable to live in a dry and sunny place, the treatment will resolve itself more particularly into the management of the digestive tract. It may be possible by this alone to procure relief. In some instances, however, and particularly in those who actually do have the gouty diathesis in addition to lithemia, it will be found necessary to prescribe remedies which favor elimination. Of these the alkalies are, unquestionably, of the greatest importance, and their effects should be furthered by liberal potations of pure water. The still waters are better than those highly gaseous. And one should not be satisfied with applying the water within ; it should be as freely applied without. The daily bath, if possible the cold bath with vigorous friction thereafter, is a good thing for the lithemic.

436 Franklin Street.

DISCUSSION.

Dr. J. W. Putnam : The three papers just read are of great value and interest. It is important that every now and then the profession should review an old subject, and see just how and where we stand today. Old views are constantly being modified. In two, if not in all three, of the papers, mention was made of lithic acid in the blood. This was at one time taught, but it is now generally accepted that it is not found free in the blood.

In considering lithemia, I have always believed that there were cases in which the cause was, as laid down by Dr. Stockton, a faulty primary digestion ; that the cases were best treated according to the rules of Dr. Stockton. I also believe there is another large class of cases in which the fault lies in a faulty disassimilation. These cases are to be treated with proper diet, exercise, massage, deep inspirations, and baths.

Many of the cases are anemic, and, in addition to the usual treatment, must have iron. Routine treatment in lithemia, I believe, is sure to result in an unnecessarily large proportion of failures.

Dr. H. E. Hayd : We are really discussing indigestion, and it matters not whether you call it lithemia, oxaluria, hypochondriasis, or many other functional troubles, they still represent faulty digestion and assimilation of food. And if we accept, as most scientific men do, that these troubles are due to a want of oxygenation of certain of the nitrogenous elements of our food, then we can understand how an excessive amount of animal food brings about this condition, and particularly if there be present some peculiarity of temperament—like the so-called gouty or rheumatic diathesis—which might be represented in the individual by a deficient digestive capacity for azotized foods. Nor does it concern us whether free lithic acid exists in the blood. The treatment of that group of symptoms which we look upon as lithemic is *oxygen*, which is best taken in the shape of active exercise. By this means the health of the whole organism is increased, and oxidation is better performed, and the various emunctories of the body eliminate their effete matters more readily. I have ceased to prescribe pepsin, drastan, pancreatine, and other ferments in these cases, as I do not believe that there is present in these persons organic changes of the stomach necessarily, but that this condition is better explained upon the grounds of hepatic torpor, resulting from indolent habits and an over-indulgence of rich foods and spirits. One would expect that vegetable foods would do best in these cases, but I don't believe it is well to enforce any special or rigid form of diet, as I have often seen vegetable foods cause so much flatulence, inaptitude for exertion, and discomfort, that exercise, which was most desirable, could not or would not be taken. And, on the other hand, I have seen many of these cases relieved by the Salsbury treatment, which consisted in the ingestion of large amounts of hot water and a free supply of raw or partly cooked animal food,—beefsteak. The physiological chemistry of this lithemic condition is interesting, and, I believe, the symptoms are much the same, whether we find an excess of urates in the urine as a precipitate after it has stood in a vessel a few hours, or whether we find free uric acid crystals. Yet, I believe, it is a more fortunate thing when there exists the excessive acid phosphate of soda to keep the uric acid in solution, as the passage of free uric acid crystals causes more bladder and urethral irritation.

Dr. F. P. Vandenbergh: I spent one whole hour this afternoon trying to explain to a lumberman what lithemia meant, and it was a difficult thing to do. We start with the albuminoids of the flesh series. These are digested imperfectly, and, as a result, we find uric acid in the urine, along with creatin, creatinine, xanthine, and other toxines. The important point, it seems to me, is, where and how do these changes take place? I don't know that anyone knows. Uric acid is not found in the blood, but is found in the urine.

The ingestion of large quantities of water flushes out the creatinine, xanthine, etc., hence it is important to bear this fact in mind in treating lithemic patients.

Dr. Allen A. Jones: I have been very much interested by the three classical papers read this evening. Dr. Krauss mentions peptone as being the final product of the gastric digestion of proteids. It is interesting to note, in this connection, the recent investigations of Drs. Gumlich and Ewald, demonstrating that, in many cases of apparently normal digestion, the proteid change, for the most part, does not go beyond the formation of propeptone. I have personally been able, in a very moderate amount of work done in this line, to demonstrate the presence of both propeptone and peptone in the gastric contents after best meals; the biuret reaction being positive, and the individual reaction for propeptone and peptone being obtained.

Dr. Pryor speaks of the great frequency with which the lithemic state occurs in the Spring. This interests me, as I have found, by the microscope, more uric acid crystals in the urine in the Spring than in any other season, and have remarked upon these seasonal changes in the urine. Phosphaturia I have found most frequently in the hot weather, while oxaluria occurs at all seasons, especially in persons with pneumatosis, nervous belching, or great gastric flatulency.

Dr. Pryor also says sodium salicylate is the best remedy for the lithemic state in which lithuria exists. This does not agree with the statement of Sir William Roberts, in a recent number of the *Lancet*, that solutions of the potassium salts dissolve more parts of uric acid per thousand than any other agent, and that next in solvent power stands plain distilled water. He shows that solutions of the sodium salts are inferior in their solvent power for uric acid than plain distilled water.

Dr. Bartlett: Among the prominent causes of lithemia may be noted deficient elimination from the cutaneous surface of the body. These excretions are often extremely toxic. Persons of sedentary habits, or whose vocation is pursued in a vitiated atmosphere, are prone to lithemia. I advise such to secure at frequent intervals abundant diaphoresis by active exercise, and, if necessary, in a heated room. Also bathing in water containing a notable amount of alkali, or of germicidal quality. As regards water in large quantity as a beverage, I think great discrimination should be used. With many, excess of water induces flatulence and painful eructations, and digestion may be greatly retarded. A patient, who had nearly completed his century, told me recently that he had been compelled to disuse it for this reason, and often passed weeks and months without so much as a glass of water. Doubtless, the soda fountains encourage injurious habits in this regard by the attractiveness of the syrups and flavors employed. Patients prone to diarrhea often aggravate the disease by yielding to inclinations to imbibe too freely, thereby increasing peristaltic action, and preventing the utilization of the elements of nutrition.

I congratulate the Academy upon the high standard of the essays read at this the initial meeting of what I confidently hope will be a long and honorable history.

---

The Buffalo Academy of Medicine will meet, after November 1, 1892, at their new parlors, 639 and 641 Main street, third floor. During November the sections will meet on the following nights:

Section on Surgery meets Tuesday evening, November 1st. President, Dr. C. C. Frederick; Secretary, Dr. W. G. Ring.

Section on Medicine meets Tuesday evening, November 8th. President, Dr. Thomas Lothrop; Secretary, Dr. H. U. Williams.

Section on Anatomy, Physiology, and Pathology meets Tuesday evening, November 15th. President, Dr. H. Mynter; Secretary, Dr. I. M. Snow.

Section on Obstetrics and Gynecology meets Tuesday evening, November 22d. President, Dr. P. W. Van Peyma; Secretary, Dr. L. C. Randall.

The next regular meeting of the Academy will be held Tuesday evening, December 6, 1892.

DELANCEY ROCHESTER, M. D., *President.*
WM. C. KRAUSS, M. D., *Secretary.*

